# Prenylated Polyphenols from *Clusiaceae* and *Calophyllaceae* with Immunomodulatory Activity on Endothelial Cells

**DOI:** 10.1371/journal.pone.0167361

**Published:** 2016-12-01

**Authors:** Caroline Rouger, Sylvain Pagie, Séverine Derbré, Anne-Marie Le Ray, Pascal Richomme, Béatrice Charreau

**Affiliations:** 1 Université d’Angers, Campus du végétal, SFR4207 QUASAV, EA921 SONAS, Beaucouzé, France; 2 INSERM UMR1064, Centre de Recherche en Transplantation et Immunologie, IHU CESTI, LabEx IGO and LabEx Transplantex, Nantes, France; 3 CHU de Nantes, Institut de Transplantation-Urologie-Néphrologie, ITUN, Nantes, France; 4 LUNAM, Université de Nantes, Faculté de Médecine, Nantes, France; Universidade do Porto, Faculdade de Farmácia, PORTUGAL

## Abstract

Endothelial cells (ECs) are key players in inflammation and immune responses involved in numerous pathologies. Although attempts were experimentally undertaken to prevent and control EC activation, drug leads and probes still remain necessary. Natural products (NPs) from Clusiaceous and Calophyllaceous plants were previously reported as potential candidates to prevent endothelial dysfunction. The present study aimed to identify more precisely the molecular scaffolds that could limit EC activation. Here, 13 polyphenols belonging to 5 different chemical types of secondary metabolites (i.e., mammea coumarins, a biflavonoid, a pyranochromanone acid, a polyprenylated polycyclic acylphloroglucinol (PPAP) and two xanthones) were tested on resting and cytokine-activated EC cultures. Quantitative and qualitative changes in the expression of both adhesion molecules (VCAM-1, ICAM-1, E-selectin) and major histocompatibility complex (MHC) molecules have been used to measure their pharmaceutical potential. As a result, we identified 3 mammea coumarins that efficiently reduce (up to >90% at 10 μM) both basal and cytokine-regulated levels of MHC class I, class II, MICA and HLA-E on EC surface. They also prevented VCAM-1 induction upon inflammation. From a structural point of view, our results associate the loss of the free prenyl group substituting mammea coumarins with a reduced cellular cytotoxicity but also an abrogation of their anti-inflammatory potential and a reduction of their immunosuppressive effects. A PPAP, guttiferone J, also triggers a strong immunomodulation but restricted to HLA-E and MHC class II molecules. In conclusion, mammea coumarins with a free prenyl group and the PPAP guttiferone J emerge as NPs able to drastically decrease both VCAM-1 and a set of MHC molecules and to potentially reduce the immunogenicity of the endothelium.

## Introduction

Located at the interface between blood and tissues, endothelial cells (ECs) lining the vasculature are key cells involved in the control of vascular homeostasis, blood pressure, coagulation, inflammation and leukocyte trafficking [1]. ECs also express classical major histocompatibility complex (MHC) class I and class II molecules which participate in the activation of immune responses [2, 3]. Moreover, human ECs express a specific set of non classical MHC molecules including MHC class I related chain A (MICA) and HLA-E that provide ECs with specific roles in both innate and adaptive immunity [4]. In response to inflammation, ECs become activated and display changes in phenotype and functions. Activated ECs favor inflammation by the expression of adhesion molecules for activated leukocytes and the release of inflammatory soluble mediators such as cytokines and chemokines. Activated ECs promote natural killer (NK), CD4+ and CD8+ T cell activation and effector functions via the upregulation of MHC and MHC-like molecules. EC activation may be transient and reversible or sustained during acute inflammation leading to endothelial cell dysfunction. Endothelial dysfunction is involved in numerous disorders, including sepsis, cardiovascular diseases and solid organ transplant rejection. Attempts to prevent and control EC activation and dysfunction can be achieved experimentally by the inhibition of signaling pathway, such as the NF-κB pathway by antioxidant compounds [*e*.*g*. pyrrolidine dithiocarbamate (PDTC)] [5, 6] as well as by apoptosis mediator [7] or effector [8] molecules. However, identification of new drug leads and targets is still required in the field.

The present study focused on the ability of polyphenols produced by *Clusiaceae* and *Calophyllaceae* plants to prevent endothelial dysfunction. Natural products (NPs) and their derivatives (NDs) represent more than 50% of drug active compounds marketed from 1981 to 2014 [9]. NPs are biosynthesized and used by living organisms for different purposes such as defense against predators or microorganisms. This could explain why NPs and NDs constitute almost the two third of anticancer, immunosuppressive, antibacterial, anticoagulant, antifungal and antiparasitic drugs [9]. NPs display a large chemodiversity [10] and play a key role in pharmaceutical research: they could be structural leads of new drugs [9] as well as a way to investigate new biological pathways and to discover new therapeutic targets [11]. We previously reported on the potential of secondary metabolites from Clusiaceous and Calophyllaceous species to reduce endothelial dysfunctions [12, 13]. These vegetal species are pantropical trees and shrubs known to biosynthesize phenol derivatives, such as coumarins, xanthones, polyprenylated polycyclic acylphloroglucinols (PPAPs) and biflavonoids [14–16]. A number of them are prenylated compounds, which often contributes to increase their biological activities [17]. Several polyphenols isolated from these families exhibit anti-inflammatory [18] or immunosuppressive [19, 20] activities and some species are used in folk medicine against inflammatory diseases [21].

Our previous investigations on Clusiaceous and Calophyllaceous species allowed us to build an in-house chemical library which contains dozens of various polyphenols with potential pharmacological activities and available for investigations in cellular models [22–24]. The present work aimed at determining their ability to modulate the expression of endothelial molecules involved in inflammation and immune responses. Monitoring EC activation *in vivo* using noninvasive approaches is still limited in humans. To select the most promising scaffolds, the inhibitory effect of natural polyphenols was thus tested on resting and cytokine-activated EC cultures. Quantitative and qualitative changes in the expression of both adhesion molecules and MHC molecules were used to establish the anti-inflammatory and immunosuppressive bioactivities of a set of natural compounds. Here, 13 molecules from 5 different chemical types of secondary metabolites (*i*.*e*. mammea coumarins, a biflavonoid, a pyranochromanone acid, a PPAP and two xanthones) were analyzed. Structure-activity relationships were further investigated for various mammea coumarins.

## Materials and Methods

### Chemicals

Coumarin (**1**) and umbelliferone (**2**) were purchased from Sigma-Aldrich (St Quentin Fallavier, France).

### The Chemical Library

**Fig 1:**
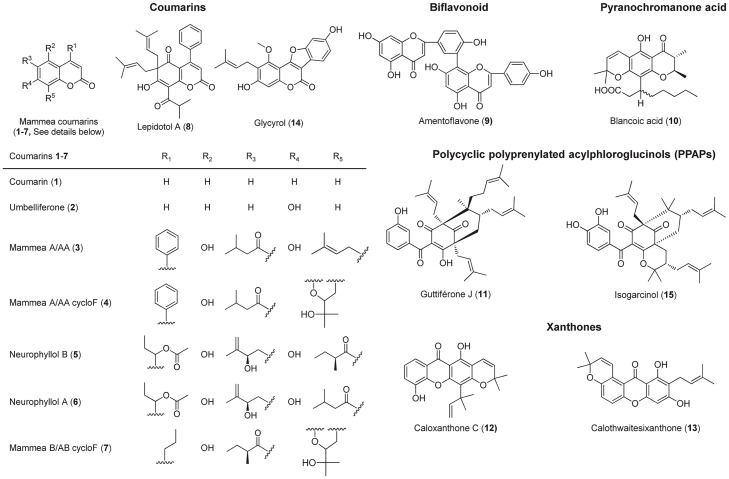
Names, structures and chemical types of mentioned NPs 1–15.

Other tested NPs were chosen from the in-house chemical library of our laboratory which includes 139 polyphenols isolated from Clusiaceous and Calophyllaceous species. The extraction and purification or synthesis procedures of mammea A/AA (**3**), mammea A/AA cycloF (**4**), neurophyllols B (**5**) and A (**6**), mammea B/AB cycloF (**7**), lepidotol A (**8**), amentoflavone (**9**), blancoic acid (**10**), guttiferone J (**11**), caloxanthone C (**12**) and calothwaitesixanthone (**13**) have been previously described [12, 13, 25–28] (Fig 1). Their purity was evaluated using high performance liquid chromatography (HPLC). HPLC analysis were performed on an Ultimate 3000 Dionex apparatus (consisting of a pumping system, a vacuum degasser, a DAD detector (Thermo Fisher Scientific, Villebon-sur-Yvette, France) and an Evaporative light scattering detector (ELSD) PL-ELS-2100 (Varian S.A., Beuvry, France) assisted by the Chromeleon software (Thermo Fisher Scientific). A 10 μL sample (1 mg/mL) was directly injected onto a Lichrospher 100 RP18 column (150 × 4.6 mm, 5 μm, Agilent Technologies, Les Ulis, France) using an acidic water (formic acid 0.1%)/acetonitrile (ACN) system. The mobile phase was as follows: Acidic water/ACN from 95:5 to 0:100 v/v in 40 min and 0:100 for 15 min. The flow rate was 1 mL/min. UV detection was achieved at 210 nm and λ_max_ for each NP. Sample purities were beyond 99.0% with ELSD except for **5**–**6** which is a mixture of neurophyllols B and A (93:7) and **11** (84.1%) (S1 Table). Stock solutions (10 mM) were prepared in dimethylsulfoxide (DMSO, Fisher Scientific, Illkirch, France) for all compounds and further diluted in the culture medium to ensure a final DMSO percentage below 0.1%.

### Reagents for Biological Assays

Glyoxal, pyrrolidine dithiocarbamate (PDTC), 3-[4,5-dimethylthiazol-2-yl]-2,5-diphenyltetrazolium bromide; thiazolyl blue (MTT) and zoledronic acid (ZA) were purchased from Sigma-Aldrich. Cyclosporin A (CsA, Sandimmun^®^ 50 mg/mL) was from Novartis Pharma SAS (Rueil-Malmaison, France). The cytokines TNF and IFNγ were purchased from R&D Systems (Lille, France).

### Endothelial Cell Culture and Activation

Primary human umbilical vein endothelial cells (HUVECs, Lonza Verviers SPRL, Verviers, Belgium) were cultured in endothelial cell basal medium (ECBM, PromoCell, Heidelberg, Germany) supplemented with 10% fetal calf serum (FCS), 4 μL/mL endothelial cell growth supplement/heparin, 0.1 ng/mL human epithelial growth factor, 1 ng/mL human basic fibroblast growth factor, 1 μg/mL hydrocortisone (C-39210, PromoCell), 2 mM glutamine, 100 U/mL penicillin and 100 μg/mL streptomycin (Life Technologies, Cergy Pontoise, France). HUVECs from 3 independent donors were used for the assays. For activation, ECs were grown to confluence in 6-well, 12-well, or 96-well plates and starved overnight in ECBM supplemented with 2% FCS before treatment. Prior addition of TNF (200 U/mL, 6h) or IFNγ (100 U/mL, 48h) cells were preincubated with NPs or controls (diluent, PDTC, ZA, CsA, glyoxal) for the indicated periods of time. Experiments were also performed without cytokines to evaluate the effect of NPs on the constitutive expression of the cell surface molecules.

### Cell Viability Assay

Cell viability was quantified by measuring metabolic activity using MTT, a water soluble tetrazolium salt, reduction assay. Briefly, HUVECs were cultured to confluence in 96-well plates and treated for 6h or 48h with either NPs (from 0.5 to 100 μM), or with controls (ZA, 10 μM; glyoxal,4 mM) in the presence or absence of TNF. Then, culture medium was removed and cells were incubated with MTT (1 mg/mL) for 3h at 37°C. The formazan crystals produced were dissolved in DMSO and the absorbance was measured at 570 nm. Assays were performed in triplicate. Percentages of cell viability were calculated using the values obtained with TNF or medium alone as 100%.

### Cellular Elisa for VCAM-1

HUVECs were plated in 96-well plates (1.10^4^ cells/well) and cultured overnight to reach confluency. Confluent monolayers were then preincubated for 1h with NPs (from 0.5 to 100 μM) or with a pharmacological inhibitor of VCAM-1 (PDTC, 200 μM) before a subsequent incubation with or without TNF (200 U/mL) for 6h. After treatment, cells were fixed and VCAM-1 proteins were detected using an anti-VCAM-1 mouse monoclonal antibody (5 μg/mL, R&D Systems) and revealed using horseradish peroxidase-labelled secondary antibodies (1 μg/mL, Cell Sciences, Inc., Canton, MA, USA). After incubation with substrate, optical density was analyzed at 405 nm. Three concentrations were tested for each NP. At this prescreening step, NPs were evaluated at 0.5, 5 and 50 μM except compounds **8** and **10** which were tested at 0.5, 5 and 50 μg/mL. Experiments were performed in triplicates and repeated in three independent assays.

### Immunostaining and Flow Cytometry

HUVECs were plated in 6 or 12-well plates (1.5.10^5^ cells/well) and cultured overnight to reach confluency. Confluent cell monolayers were then preincubated for 1h with NPs (10 μM) or controls (PDTC 200 μM; CsA 10 μM) or 18h with ZA (10 μM) before addition of TNF (200 U/mL for 6h) or IFNγ (100 U/mL for 48h). Cells were immunostained using anti-VCAM-1, anti-E/P-selectin, anti-ICAM-1-FITC (R&D Systems), anti-pan HLA class I (anti-HLA-A, -B and -C; clone W6/32), anti-pan HLA class II (anti-HLA-DR, -DP, -DQ; clone L243), anti-MICA (clone AMO1) (BamOmab, Tubingen, Germany), anti-HLA-E-APC (clone 3D12) (Miltenyi Biotec, Paris, France) mouse IgG and anti-mouse IgG+IgM (H+L)-FITC (Jackson Immunoresearch Laboratories, West Grove, PA, USA) antibodies at 10 μg/mL or diluted at 1/100. An isotype-matched IgG was used as a non-specific control and binding of antibodies to the cells was evaluated by flow cytometry using 10 000 cells/sample and a BD FACSCanto^™^ II flow cytometer (Becton Dickinson, San Jose, CA, USA). Cells were gated on forward scattered light (FSC) *versus* side scattered light (SSC) parameters to select live cells. Data were analyzed using FlowJo software (Tree Star, Inc., Ashland, OR, USA) and depicted in histograms plotting geometric mean of fluorescence intensity (GFI) on a four-decade logarithmic scale (x-axis) versus cell number (y-axis).

### Statistical Analysis

At least three independent experiments were performed for each condition. Data were expressed as means ± SD and compared using the nonparametric ANOVA or Kruskal-Wallis test with Dunn’s multiple comparison post-test. A *p* value of ≤ 0.05 was considered significant. One, two and three asterisks (*, ** and ***) denote *p* < 0.05, 0.01 and 0.001, respectively.

## Results

### Selection of Natural Polyphenols from *Clusiaceae* and *Calophyllaceae* Plants

Thirteen NPs (**1–13**) associated with 5 different chemical types of Clusiaceous and Calophyllaceous secondary metabolites were selected for the study (Fig 1). They were chosen in our in-house chemical library considering their structure, purity (S1 Table) and availability. NPs **1–13** include a set of mammea coumarins (**1**–**8**), a biflavonoid (**9**), a pyranochromanone acid (**10**), a PPAP (**11**) and two xanthones (**12**–**13**) (Fig 1)**.** A particular focus was given to mammea coumarins (**3**–**8**, R_2_ = R_4_ = OH or O) isolated from *Clusiaceae* and *Calophyllaceae* plants, previously described as promising compounds in this field [26, 29]. Their structure-activity relationships were more thoroughly investigated here. Coumarins were chosen in the library according to their substituents: R_1_ is either a phenyl (**3**–**4**,**8**), a 1-(acetoxy)-propyl (**5**–**6**) or a propyl (**7**) group; these coumarins carry an acyl in R_3_ and a prenyl in R_5_ (**3**–**4**,**7**) or *vice versa* (**5**–**6**,**8**); finally, the prenyl group may be cyclized (**4**,**7**) or not (**3**,**5**–**6**,**8**). To investigate the bioactive potential of mammea coumarins in comparison with simple derivatives, coumarin (**1**) and umbelliferone (**2**) were also tested.

### Screening Based on Cytotoxic Activity and Inhibition of VCAM-1

Firstly, as a screening step, NPs **1**–**13** were tested for cytotoxicity toward EC cultures and for their ability to inhibit TNF-induced expression of the adhesion molecule VCAM-1 on cell surface. To this aim, EC monolayers were incubated with TNF in the presence of NPs. PDTC, a potent inhibitor of NF-κB [5] which is a transcription factor involved in VCAM-1 expression, was used as a positive control. After treatment, cell viability was assessed using a MTT assay while, in parallel experiments, VCAM-1 expression was revealed using an indirect two-step immunostaining protocol. The activity of NPs on cell viability and expression of VCAM-1 was tested at 3 concentrations (ranging from 0.5 to 100 μM). As a result, natural xanthones, *i*.*e*. caloxanthone C (**12**) and calothwaitesixanthone (**13**), were associated with a reduced, although not statistically significant, cell viability supportive of a cytotoxic effect towards ECs and thus were not investigated further. The other NPs appear either nontoxic or displayed a moderate (<25%) toxicity only at the higher concentration tested (50–100 μM) (S1A Fig). Unexpectedly, NPs **1**, **3**, **5**/**6**, **7**, **9** and **11** display values of cell viability over 100% that may suggest either a protective effect compared to control conditions or a nonspecific interference with the metabolic pathway measured by the MTT assay. Our results show that most of the NPs exhibited an inhibitory activity toward VCAM-1 in response to TNF ranging from 20% to 60% (S1B Fig). The more potent inhibitory effect was found for coumarin mammea A/AA (**3**). Decrease in VCAM-1 expression observed for compounds **12** and **13** was nonspecific and reflected the toxicity of NPs **12** and **13** for ECs. Cyclized cycloF mammea coumarins (**4** and **7**) exhibited identical and only faint activities. Consequently, only one of them, mammea A/AA cycloF (**4**), was included in the following steps of investigation.

### Guttiferone J and Mammea Coumarins Inhibit the Vascular Adhesion Molecule VCAM-1 on Endothelial Cells upon Inflammation

**Fig 2:**
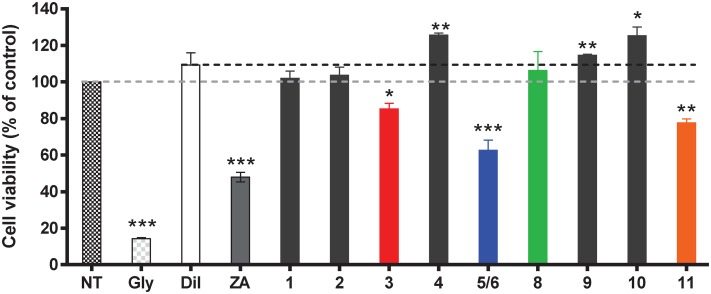
Effect of NPs 1–6 and 8–11 on endothelial cell viability. Cell viability was assessed on confluent EC monolayers using MTT assay. Cells were incubated with NPs (10 μM) for 48h before analysis. Diluted DMSO (1/1000 –dark dashed line) was used as control for diluent (Dil), as well as a non-treated (NT) control—grey dashed line—and the cytotoxicity positive control glyoxal (Gly) at 4 mM. The cytotoxic activity of the immunosuppressive drug zoledronic acid (ZA, 10 μM) was also assessed. Statistical analysis of values (O.D.) obtained for treated versus non-treated cells were performed using non-parametric ANOVA test.

At the end of the screening step, 10 natural polyphenols (**1–6**, **8–11**) were selected for further characterization. All NPs were tested at a single concentration (10 μM) to ensure comparison and avoid excessive toxicity. Toxicity of the compounds for ECs cultures at the selected concentration (10 μM) was first defined (Fig 2). Simple coumarins **1–2** had no toxic effects on ECs whereas the mammea coumarins **3**–**8** exhibited variable cytotoxicity. The prenylated coumarins mammea A/AA (**3**) and neurophyllol B (**5**)/neurophyllol A (**6**) (93/7 mixture) decreased the cell viability at 10 μM, whereas no reduction in cell viability was observed for mammea A/AA cycloF (**4**) and lepidotol A (**8**), This result may suggest a “protective effect” of the cyclization occurring between the substituents R_4_ and R_5_ in mammea coumarins, which is consistent with previous reports [30]. Amentoflavone (**9**) and blancoic acid (**10**) did not induce cell toxicity. Finally, similarly to coumarin mammea A/AA (**3**) and neurophyllols (**5**–**6**), the PPAP guttiferone J (**11**) also impacted the cell viability.

**Fig 3:**
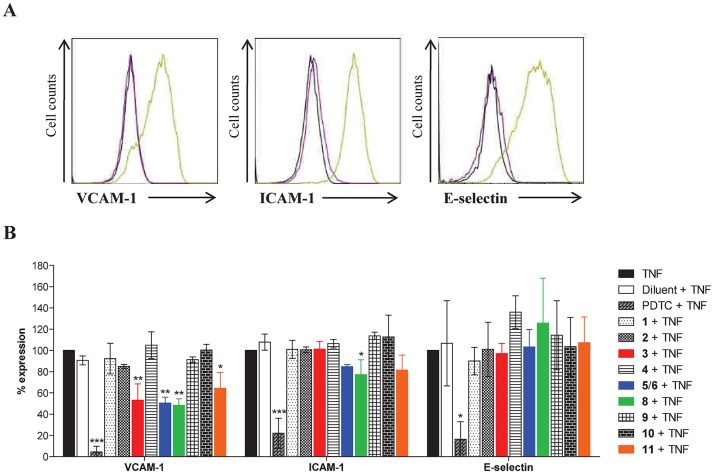
Effect of compounds 1–6 and 8–11 on TNF-induced expression of inflammatory molecules in ECs. **(A) Basal *versus* TNF-induced expression of VCAM-1, ICAM-1 and E-selectin after 6h of incubation.** Data shown are representative histograms from fluorescence-activated cell sorting (Facs) analysis showing constitutive *versus* induced expression of inflammatory molecules (VCAM-1, ICAM-1, E-selectin) at the cell surface of ECs. Histograms show the intensity of fluorescence (log, x-axis) versus cell number (y-axis) for untreated (purple line) and cytokine-treated (green line) ECs analyzed by flow cytometry. Immunostaining using an irrelevant isotype-matched IgG (dark line) was used as a negative control. (**B**) **Inhibitory effects of NPs.** Expression of VCAM-1, ICAM-1 and E-selectin was measured by Facs. Each bar represents geometric mean ± SD of fluorescence intensity calculated from three to five independent experiments. Statistical analysis was performed using Kruskal-Wallis test with Dunn’s post-test; *p < 0.05, **p < 0.01, ***p < 0.001 compared with TNF control group. PDTC (200 μM) was used as a positive control for inhibition.

Next, qualitative and comparative analyses were performed using flow cytometry. In comparison with cellular ELISA, flow cytometry allows the relative quantification of several markers on the same cell and a selective gating on live cells. In our study, the analysis of 3 inflammatory markers (VCAM-1, ICAM-1, E-selectin) was achieved. As depicted on Fig 3A, EC cultures don’t express basally VCAM-1, ICAM-1 or E-selectin. These inducible adhesion molecules are strongly upregulated in response to TNF (Fig 3A). Consequently, the activity of the simple coumarins **1**–**2** as well as the Clusiaceous and Calophyllaceous polyphenols **3**–**6** and **8**–**11** was assessed by measuring the surface-expression of inflammatory mediators induced by TNF. Our findings (Fig 3B) revealed that active prenylated polyphenols especially inhibited VCAM-1 expression. A PPAP, guttiferone J (**11**) and some mammea coumarins, *i*.*e*. **3**, **5**/**6** (93/7) and **8**, significantly suppressed VCAM-1 expression when compared to TNF alone. On the other hand, simple coumarins **1**–**2** and mammea A/AA cycloF (**4**) did not exhibit inhibitory activity, suggesting that substitution of the coumarin skeleton was a necessary condition for anti-inflammatory activity, whereas an additive cyclization between R_4_ and R_5_ in mammea coumarins cancelled this inhibitory potential. Expression of ICAM-1 was only significantly decreased (23%) by coumarin **8**, whereas other tested NPs did not affect E-selectin expression. At 10 μM, amentoflavone (**9**), a common biflavonoid in Clusiaceous and Calophyllaceous species and blancoic acid (**10**), a pyranochromanone acid, did not modulate the expression of the inflammatory markers. Experiments were also performed without TNF to exclude any potential proinflammatory effect. No increase in the basal expression was observed in presence of the tested NPs indicating no pro-inflammatory activity for these polyphenols (S2 Fig).

### Guttiferone J and Mammea Coumarins are Potent Inhibitors of MHC Expression

**Fig 4:**
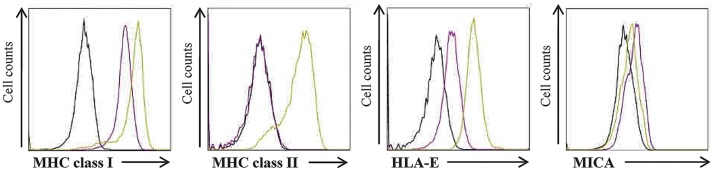
Basal *versus* IFNγ-modulated expression of MHC class I, MHC class II, HLA-E and MICA on EC cultures. ECs were treated with or without IFNγ for 48h before analysis. Data shown are representative histograms from Facs analysis showing constitutive *versus* IFNγ-regulated expression of inflammatory molecules (MHC class I, MHC class II, HLA-E and MICA) at the EC surface. Histograms show the intensity of fluorescence (log, x-axis) *versus* cell number (y-axis) for untreated (purple line) and cytokine-treated (green line) ECs analyzed by flow cytometry. Immunostaining using an irrelevant isotype-matched IgG (dark line) was used as a negative control.

ECs basally coexpress a broad array of MHC molecules including the MHC-like molecules MICA and HLA-E [4] as illustrated in the Fig 4. Consequently, ECs are suitable candidates for drug testing strategy aiming at identifying bioactive compounds with either broad or selective effects on leukocyte adhesion and/or leukocyte activating immune molecules. Importantly, whereas inflammatory molecules are responsive to TNF, MHC class I (HLA-A,-B,-C) and class II (HLA-DR, DP, -DQ) molecules are upregulated by the cytokine IFNγ, with the exception of MICA which is downregulated by IFNγ [31] (Fig 4).

**Fig 5:**
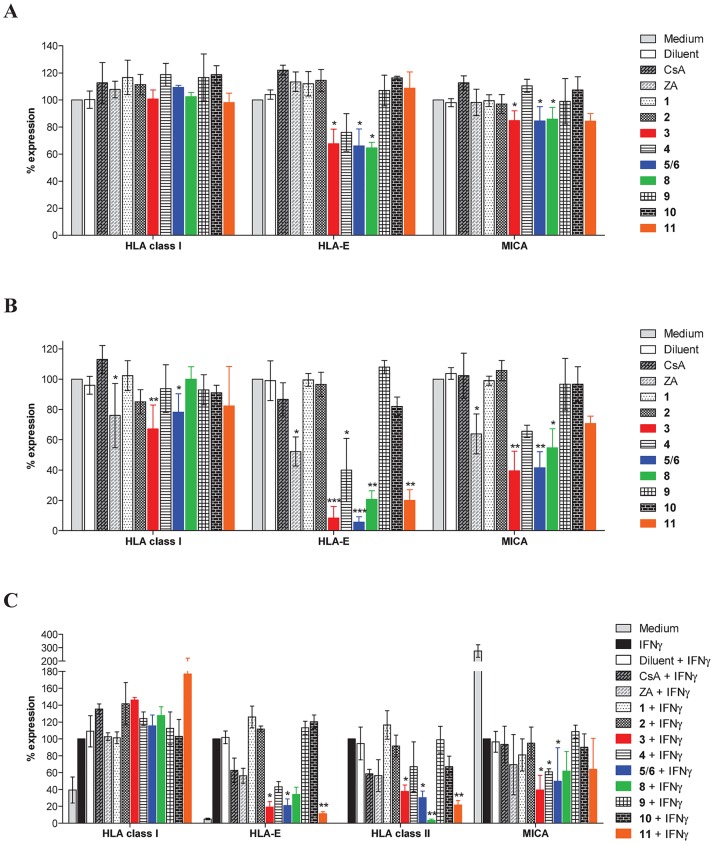
Immunomodulatory activities of NPs in ECs targeting MHC molecules. (**A**) Regulatory effects of compounds **1**–**6** and **8**–**11** (10 μM) on the basal expression of MHC molecules after 1h of incubation. (**B**) Regulatory effects of compounds **1**–**6** and **8**–**11** (10 μM) on the basal expression of MHC molecules after 48h of incubation. (**C**) Regulatory effects of compounds **1**–**6** and **8**–**11** (10 μM) on the expression of MHC molecules regulated by IFNγ for 48h. Cyclosporin (CsA, 10 μM) and zoledronic acid (ZA, 10 μM) were used as immunosuppressive references. For all conditions, cells were preincubated for 1h with NPs or CsA or for 18h with ZA. Expression of MHC molecules was measured by Facs. Each bar represents geometric mean ± SD of fluorescence intensity from three to five independent experiments. Statistical analysis was performed using Kruskal-Wallis test with Dunn’s post-test; *p < 0.05, **p < 0.01, ***p < 0.001 compared to controls (medium or IFNγ).

In this study, two reference compounds were used as controls: the bisphosphonate zoledronic acid (ZA) previously reported as acting on immunity [32] and the immunosuppressive drug and calcineurin inhibitor cyclosporin A (CsA) [33]. Since basal levels of MHC class I and MHC class I-like (HLA-E and MICA) are detected on EC surface, the immunomodulatory potential of NPs **1**–**6** and **8**–**11** was first studied by measuring their effect on the constitutive surface-expression of MHC molecules after a short (1h) or a long term (48h) treatment. After 1h (Fig 5A**)**, we observed that only mammea coumarins **3**, **5**/**6** and **8** had a slight but significant inhibitory effect on basal HLA-E (30% of inhibition) and MICA (<20%). No effect was found for MHC class I molecules. Their regulatory effect was enhanced after 48h of treatment (Fig 5B) with a major effect on HLA-E (up to 90% of inhibition) and MICA (up to 60% of inhibition) while a slight but significant effect was also observed for MHC class I molecules for mammea coumarins **3** and **5/6**. After 48h (Fig 5B), ZA, the reference compound, significantly inhibited MHC class I, HLA-E and MICA on ECs. Interestingly, mammea A/AA cyclo F (**4**) seemed also effective although inhibitions were not found significant except for HLA-E at 48h. In addition to these effects observed for mammea coumarins, we observed that the PPAP, guttiferone J (**11**), also strongly reduced HLA-E level at 48h.

MHC class I, class II and HLA-E molecules are readily upregulated on ECs in response to IFNγ (Fig 5C). In these conditions, ZA and the tested polyphenols were not able to reduce the upregulation of MHC class I molecules. ZA displayed no significant effect on HLA-E, MICA and MHC class II in the presence of IFNγ although a trend toward inhibition was observed. In contrast, mammea coumarins **3**, **5**/**6** and **8** and guttiferone J (**11**) were potent inhibitors of MHC class II induction mediated by IFNγ. Moreover, mammea coumarins and guttiferone J were also potent inhibitors of the upregulation of HLA-E by IFNγ that preserve HLA-E level close to the basal level. Interestingly, mammea coumarins **3**, **4** and **5**/**6** potentiate the inhibition of MICA in response to IFNγ, leading to an overall >90% decrease compare to basal level.

## Discussion

**Fig 6:**
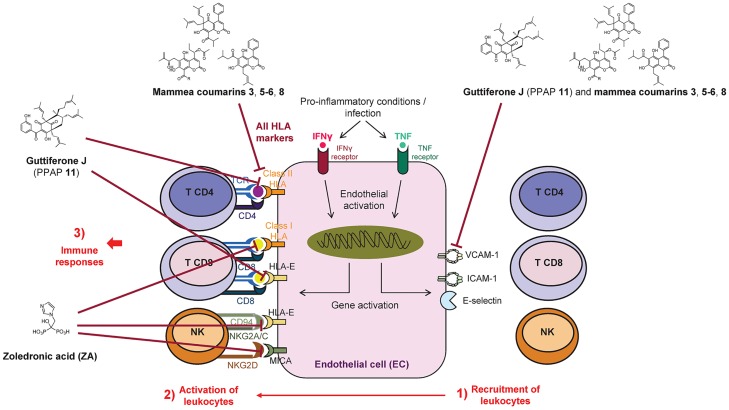
Prenylated polyphenols from *Clusiaceae* and *Calophyllaceae* as regulators of inflammation and immunity: Overview and hypothesis. (1) NPs efficiently inhibit the induction of VCAM-1, an adhesion molecule involved in the firm adhesion of leukocytes on inflamed endothelium and a prerequisite step for leukocyte activation (2) and extravasation in the tissues. (3) NPs strongly impair the expression of MHC molecules expressed on endothelium and involved in innate and adaptive immunity via the activation of NK and CD4T and CD8T cells, respectively.

Among thirteen polyphenols tested during this study, mammea coumarins and PPAPs such as guttiferone J (**11**), usually isolated from Clusiaceous and Calophyllaceous species, emerged as NPs with a molecular scaffold able to modulate expression of immune as well as inflammation markers on ECs. In particular, a major result from this study is the profile of markers inhibited by the mammea coumarins: Coumarins **3**, **5/6**, and **8** efficiently reduce both basal and cytokine-regulated levels of MHC class I and class II, MICA and HLA-E on EC surface up to >90% for MHC class II, HLA-E and MICA. The molecular scaffold of PPAPs emerges from the study as well: Guttiferone J (**11**) also triggers a strong immunomodulation but restricted to HLA-E and MHC class II molecules. The concomitant inhibition of VCAM-1, the ligand for VLA4 on activated leukocytes, suggests that both mammea coumarins and guttiferone J act on two steps of EC/leukocyte interaction (1) by preventing the firm adhesion of activated leukocytes on the endothelium, a prerequisite step for extravasation in the tissue and (2) by inhibiting the direct interaction with a set of immunoreceptors αβ T cell receptor cells, NKG2D and CD94/NKG2A/C (Fig 6).

In contrast to MHC class I, MHC class II, HLA-E and MICA are expressed only by a restricted set of cells which includes mostly cells involved in immunity such as antigen presenting cells (ECs, monocytes, dendritic cells) and lymphocytes [34, 35]. Among them, ECs coexpress MHC class I, MHC class II, HLA-E and MICA. MHC class I and class II are masterpieces of adaptive immune responses triggering antigen-specific CD8+ and CD4+ T cell activation, respectively. HLA-E and MICA play more complex roles as ligands for inhibitor and activating receptors (CD94/NKG2A and NKG2D, respectively) providing control for NK cells activation and costimulation of CD8+ T cells [4, 36, 37]. MICA and HLA-E have key functions in the immune responses against cancer, infection and allogeneic solid organ transplant [38]. Moreover, MICA and HLA-E also play different or even opposite role according to the pathologic context. Consequently, for the moment, further studies are required to determine whether the reduction of MHC can be achieved *in vivo* and in which model/pathology it could provide clinical benefit. Mammea coumarin as well as PPAPs selected in this work could help achieve this goal.

Only statins were previously reported to decrease MHC class II molecules, a process due to inhibition of the inducible promoters I and IV of the transactivator CIITA and observed in several cell types, including primary human ECs and monocyte-macrophages [39]. In contrast, the effect of statins on MHC class I remains controversy probably due to both cell type-specific and statin-specific effects. Statins are well-known hydroxy-methylglutaryl-CoA (HMG-CoA) reductase inhibitors which prevent the biosynthesis of cholesterol but also the production of isoprenoid intermediates such as geranyl-geranyl pyrophosphate (GGPP) and farnesyl pyrophosphate (FPP). GGPP and FPP are crucial for the post-translational modifications of proteins such as the small GTP-binding proteins Ras, Rac, and Rho. [40]. As this isoprenylation step is essential for various cellular functions, the inhibition of the mevalonate pathway by statins could explain their pleiotropic effects [8]. Statins potentiate IFNγ-induced MHC class I expression in tumor cell lines [41] but reduce both basal and inducible expression in primary muscle cell line [42]. In human ECs, while the initial study from Kwak *et al* demonstrated no effect [39], Belliard *et al* reported that some statins upregulate IFNγ-induced MHC class I [43]. Although the mechanism remain unclear, statins might affect cytokine induced expression of STAT1 and the expression of proteins involved in class I antigen presentation (TAP and LMP) [42]. Statins were also found to increase MICA expression on melanoma cells *in vitro* and melanoma growth as well as pulmonary metastases in mice [44].

Although preliminary, our findings may also reveal a new role for the bisphosphonate ZA in immune modulation through a regulatory action of a set of MHC molecules including MHC class I, HLA-E and MICA. In our conditions, the effect of ZA appeared for *in vitro* long term (48h) treatment suggesting that ZA may affect protein turn over and/or *de novo* synthesis. ZA belongs to aminobisphosphonates (NBPs), an important class of molecules with different molecular mechanisms of action used in the treatment of bone diseases [45]. ZA is a very potent inhibitor of farnesyl-pyrophosphate synthase (FPP), an enzyme acting downstream from HMG-CoA reductase, the rate-limiting enzyme in the mevalonate pathway. Recently, immunomodulation of γδ T lymphocytes has been reported for ZA associated with anti-tumor activity [32] in addition to its previously described anti-angiogenic, anti-metalloproteinase or pro-apoptotic activities [45]. From the best of our knowledge, our study provides the first description of an immunomodulation targeting MHC expression for NBPs. Nevertheless, at equal concentration, our results established mammea coumarins **3**, **5**/**6** and **8** as well as the PPAP guttiferone J (**11**) as more potent MHC modulators when compared with ZA.

Mechanism of MHC regulation mediated by the prenylated polyphenols investigated here still remains to be elucidated. Mammea coumarins were found active whereas simple coumarins were not. A substituent at C-4 appeared to be a necessary condition for activity, however the nature of this substituent (*i*.*e*. phenyl, alkyl, 1-acetoxypropyl) seems to have no influence on the activity. Although cyclized mammea coumarins such as mammea A/AA cycloF (**4**) are less cytotoxic for ECs, the cyclization seems to reduce their activities. The presence of a prenyl substituent oxidized or not, thus appeared as essential. Previously glycyrol (**14**), another prenylated coumarin, was described as a calcineurin inhibitor and also emerged as a potential immunomodulatory drug [29, 46]. Similar effects on both immune and inflammatory mediators were observed with the PPAP **11** as well. Isogarcinol (**15**), another PPAP was recently described as a potent immunosuppressive agent acting as a calcineurin inhibitor [19, 47], suggesting that a structure-activity relationship analysis of a larger panel of PPAPs would probably be useful.

In conclusion, prenylated coumarins exhibiting a prenyl substituent and the PPAP guttiferone J (**11**) show anti-inflammatory and immunosuppressive activities by reducing the expression of a panel of molecules on EC surface. These findings could suggest a common mechanism of regulation mediated by these NPs and a high therapeutic value for these natural prenylated polyphenols. Such secondary metabolites from Clusiaceous and Calophyllaceous species thus appear as promising model compounds for the development of new immunomodulatory drugs.

## Supporting Information

S1 Table Purity (%) of NPs from the chemical library evaluated by HPLC equipped with ELSD and UV detectors. (DOCX)

S1 Fig Effects of the polyphenolic compounds on EC viability and VCAM-1 expression. **(A)** Cellular toxicity on ECs was evaluated by a MTT assay using glyoxal (4 mM) as inducer of cell death control. **(B)** VCAM-1 surface expression was assessed by a cellular ELISA assay after activation of the cells by TNF (200 U/mL, 6h) and using pyrrolidine dithiocarbamate (PDTC, 200 μM) as an inhibitory reference. Statistical analysis of values (O.D.) obtained for treated *versus* non-treated cells (A) or TNF (B) were performed using non-parametric ANOVA test; (*p < 0.05, **p < 0.01 *versus* TNF).
(TIF)

S2 Fig Effect of NPs 1–6 and 8–11 on the basal expression of inflammatory molecules in ECs. ECs were incubated with NPs (10 μM) for 6h in the absence of TNF. Cells were analyzed by facs for the expression of VCAM-1, ICAM-1 and E-selectin. TNF was used as a positive control.
(TIF)

## References

[1] Aird WC. Endothelium in health and disease. Pharmacol Rep. 2008;60(1):139–43. 18276995

[2] Choi J, Enis DR, Koh KP, Shiao SL, Pober JS. T lymphocyte-endothelial cell interactions. Annu Rev Immunol. 2004;22:683–709. Epub 2004/03/23. 10.1146/annurev.immunol.22.012703.104639 15032593

[3] Pober JS, Tellides G. Participation of blood vessel cells in human adaptive immune responses. Trends Immunol. 2012;33(1):49–57. 10.1016/j.it.2011.09.006 22030237 PMC3253953

[4] Gavlovsky PJ, Tonnerre P, Guitton C, Charreau B. Expression of MHC class I-related molecules MICA, HLA-E and EPCR shape endothelial cells with unique functions in innate and adaptive immunity. Hum Immunol. 2016. 10.1016/j.humimm.2016.02.007 26916837

[5] Khachigian LM, Collins T, Fries JW. N-acetyl cysteine blocks mesangial VCAM-1 and NF-kappa B expression in vivo. Am J Pathol. 1997;151(5):1225–9. 9358747 PMC1858066

[6] Cook-Mills JM, Marchese ME, Abdala-Valencia H. Vascular Cell Adhesion Molecule-1 expression and signaling during disease: Regulation by reactive oxygen species and antioxidants. Antioxid Redox Signal. 2011;15(6):1607–38. 10.1089/ars.2010.3522 21050132 PMC3151426

[7] Zhang Y, Pan Y, Bian Z, Chen P, Zhu S, Gu H, et al Ceramide production mediates aldosterone-induced human umbilical vein endothelial cell (HUVEC) damages. PLoS One. 2016;11(1):e0146944 10.1371/journal.pone.0146944 26788916 PMC4720365

[8] Bedi O, Dhawan V, Sharma PL, Kumar P. Pleiotropic effects of statins: new therapeutic targets in drug design. Naunyn-Schmiedeberg's Arch Pharmacol. 2016;389(7):695–712. 27146293 10.1007/s00210-016-1252-4

[9] Newman DJ, Cragg GM. Natural products as sources of new drugs from 1981 to 2014. J Nat Prod. 2016;79(3):629–61. 10.1021/acs.jnatprod.5b01055 26852623

[10] Dobson CM. Chemical space and biology. Nature. 2004;432(7019):824–8. 10.1038/nature03192 15602547

[11] Schmitt EK, Hoepfner D, Krastel P. Natural products as probes in pharmaceutical research. J Ind Microbiol Biotechnol. 2016;43(2–3):249–60. 10.1007/s10295-015-1691-9 26438431

[12] Ferchichi L, Derbré S, Mahmood K, Touré K, Guilet D, Litaudon M, et al Bioguided fractionation and isolation of natural inhibitors of advanced glycation end-products (AGEs) from *Calophyllum flavoramulum*. Phytochemistry. 2012;78(0):98–106. 22445651 10.1016/j.phytochem.2012.02.006

[13] Dang BT, Geny C, Blanchard P, Rouger C, Richomme P, Seraphin D, et al Advanced glycation inhibition and protection against endothelial dysfunction induced by coumarins and procyanidins from *Mammea neurophylla*. Fitoterapia. 2014;96:65–75. 10.1016/j.fitote.2014.04.005 24731922

[14] Cechinel FV, Meyre-Silva C, Niero R. Chemical and pharmacological aspects of the genus *Calophyllum*. Chemistry & Biodiversity. 2009;6(3):313–27. 19319867 10.1002/cbdv.200800082

[15] Magadula JJ. Bioactive mammea-type coumarins and benzophenones from two *Clusiaceae* plants. J Pharm Sci Innovation. 2012;1(5):31–3.

[16] Dang BT, Guitton Y, Freuze I, Grovel O, Litaudon M, Richomme P, et al Dereplication of *Mammea neurophylla* metabolites to isolate original 4-phenylcoumarins. Phytochem Lett. 2015;11:61–8.

[17] Yazaki K, Sasaki K, Tsurumaru Y. Prenylation of aromatic compounds, a key diversification of plant secondary metabolites. Phytochemistry. 2009;70(15–16):1739–45. 10.1016/j.phytochem.2009.08.023 19819506

[18] Dzoyem JP, Lannang AM, Fouotsa H, Mbazoa CD, Nkengfack AE, Sewald N, et al Anti-inflammatory activity of benzophenone and xanthone derivatives isolated from *Garcinia* (*Clusiaceae*) species. Phytochem Lett. 2015;14:153–8.

[19] Cen J, Shi M, Yang Y, Fu Y, Zhou H, Wang M, et al Isogarcinol is a new immunosuppressant. PLoS One. 2013;8(6):e66503 10.1371/journal.pone.0066503 23785505 PMC3681756

[20] Dang BT, Rouger C, Litaudon M, Richomme P, Séraphin D, Derbré S. Identification of minor benzoylated 4-phenylcoumarins from a *Mammea neurophylla* bark extract. Molecules. 2015;20(10):17735–46. 10.3390/molecules201017735 26404214 PMC6332034

[21] de Melo MS, Quintans JdS, Araujo AA, Duarte MC, Bonjardim LR, Nogueira PC, et al A systematic review for anti-inflammatory property of *Clusiaceae* family: a preclinical approach. Evid Based Complement Alternat Med. 2014;2014:960258 10.1155/2014/960258 24976853 PMC4058220

[22] Morel C, Dartiguelongue C, Youhana T, Oger J-M, Seraphin D, Duval O, et al New coumarins from *Mesua racemosa*: isolation and synthesis. Heterocycles. 1999;51(9):2183–91.

[23] Guilet D, Helesbeux J-J, Seraphin D, Sevenet T, Richomme P, Bruneton J. Novel Cytotoxic 4-Phenylfuranocoumarins from *Calophyllum dispar*. J Nat Prod. 2001;64(5):563–8. 11374944 10.1021/np000517o

[24] Gomez-Verjan J, Gonzalez-Sanchez I, Estrella-Parra E, Reyes-Chilpa R. Trends in the chemical and pharmacological research on the tropical trees *Calophyllum brasiliense* and *Calophyllum inophyllum*, a global context. Scientometrics. 2015;105(2):1019–30. 10.1007/s11192-015-1715-2 PMC7089286 32214549

[25] Raad I, Terreux R, Richomme P, Matera E-L, Dumontet C, Raynaud J, et al Structure-activity relationship of natural and synthetic coumarins inhibiting the multidrug transporter P-glycoprotein. Bioorg Med Chem. 2006;14(20):6979–87. 10.1016/j.bmc.2006.06.026 16824763

[26] Rouger C, Derbre S, Charreau B, Pabois A, Cauchy T, Litaudon M, et al Lepidotol A from *Mesua lepidota* inhibits inflammatory and immune mediators in human endothelial cells. J Nat Prod. 2015;78(9):2187–97. 10.1021/acs.jnatprod.5b00222 26301802

[27] Merza J, Mallet S, Litaudon M, Dumontet V, Seraphin D, Richomme P. New cytotoxic guttiferone analogues from *Garcinia virgata* from New Caledonia. Planta Med. 2006;72(1):87–9. 10.1055/s-2005-873185 16450306

[28] Hay A-E, Helesbeux J-J, Duval O, Labaied M, Grellier P, Richomme P. Antimalarial xanthones from *Calophyllum caledonicum* and *Garcinia vieillardii*. Life Sci. 2004;75:3077–85. 10.1016/j.lfs.2004.07.009 15474559

[29] Li J, Tu Y, Tong L, Zhang W, Zheng J, Wei Q. Immunosuppressive activity on the murine immune responses of glycyrol from *Glycyrrhiza uralensis via* inhibition of calcineurin activity. Pharm Biol. 2010;48(10):1177–84. 10.3109/13880200903573169 20860439

[30] Reyes-Chilpa R, Estrada-Muniz E, Ramirez AT, Amekraz B, Aumelas A, Jankowski CK, et al Cytotoxic effects of mammea type coumarins from *Calophyllum brasiliense*. Life Sci. 2004;75(13):1635–47. 10.1016/j.lfs.2004.03.017 15261767

[31] Chauveau A, Tonnerre P, Pabois A, Gavlovsky PJ, Chatelais M, Coupel S, et al Endothelial cell activation and proliferation modulate NKG2D activity by regulating MICA expression and shedding. J Innate Immun. 2014;6(1):89–104. 10.1159/000351605 23860405 PMC6784110

[32] Muraro M, Mereuta OM, Carraro F, Madon E, Fagioli F. Osteosarcoma cell line growth inhibition by zoledronate-stimulated effector cells. Cellular Immunol. 2007;249(2):63–72. 18163982 10.1016/j.cellimm.2007.11.005

[33] Survase SA, Kagliwal LD, Annapure US, Singhal RS. Cyclosporin A—A review on fermentative production, downstream processing and pharmacological applications. Biotechnol Adv. 2011;29(4):418–35. 10.1016/j.biotechadv.2011.03.004 21447377

[34] Zwirner NW, Fernandez-Vina MA, Stastny P. MICA, a new polymorphic HLA-related antigen, is expressed mainly by keratinocytes, endothelial cells, and monocytes. Immunogenetics. 1998;47(2):139–48. 9396860 10.1007/s002510050339

[35] Coupel S, Moreau A, Hamidou M, Horejsi V, Soulillou JP, Charreau B. Expression and release of soluble HLA-E is an immunoregulatory feature of endothelial cell activation. Blood. 2007;109(7):2806–14. 10.1182/blood-2006-06-030213 17179229

[36] Braud VM, Allan DS, O'Callaghan CA, Soderstrom K, D'Andrea A, Ogg GS, et al HLA-E binds to natural killer cell receptors CD94/NKG2A, B and C. Nature. 1998;391(6669):795–9. 10.1038/35869 9486650

[37] Zou Y, Stastny P. Role of MICA in the immune response to transplants. Tissue Antigens. 2010;76(3):171–6. 10.1111/j.1399-0039.2010.01527.x 20696027

[38] Schrambach S, Ardizzone M, Leymarie V, Sibilia J, Bahram S. In vivo expression pattern of MICA and MICB and its relevance to auto-immunity and cancer. PLoS One. 2007;2(6):e518 10.1371/journal.pone.0000518 17565371 PMC1885219

[39] Kwak B, Mulhaupt F, Myit S, Mach F. Statins as a newly recognized type of immunomodulator. Nat Med. 2000;6(12):1399–402. 10.1038/82219 11100127

[40] Greenwood J, Steinman L, Zamvil SS. Statin therapy and autoimmune disease: from protein prenylation to immunomodulation. Nat Rev Immunol. 2006;6(5):358–70. 10.1038/nri1839 16639429 PMC3842637

[41] Tilkin-Mariame AF, Cormary C, Ferro N, Sarrabayrouse G, Lajoie-Mazenc I, Faye JC, et al Geranylgeranyl transferase inhibition stimulates anti-melanoma immune response through MHC Class I and costimulatory molecule expression. FASEB J. 2005;19(11):1513–5. 10.1096/fj.04-3482fje 15990392

[42] Singh P, Kohr D, Kaps M, Blaes F. Influence of statins on MHC class I expression. Ann N Y Acad Sci. 2009;1173:746–51. 10.1111/j.1749-6632.2009.04646.x 19758224

[43] Belliard G, Coupel S, Charreau B. [Fluvastatin affects HLA class I expression on endothelial cells]. Nephrol Ther. 2005;1(4):221–7. 10.1016/j.nephro.2005.06.008 16895688

[44] Pich C, Teiti I, Rochaix P, Mariame B, Couderc B, Favre G, et al Statins Reduce Melanoma Development and Metastasis through MICA Overexpression. Front Immunol. 2013;4:62 10.3389/fimmu.2013.00062 23493799 PMC3595569

[45] Russell RG, Watts NB, Ebetino FH, Rogers MJ. Mechanisms of action of bisphosphonates: similarities and differences and their potential influence on clinical efficacy. Osteoporos Int. 2008;19(6):733–59. 10.1007/s00198-007-0540-8 18214569

[46] Peng L, Qi Y, Wu H, Wei Q. Interaction of glycyrol with calcineurin A studied by spectroscopic methods and docking. IUBMB Life. 2011;63(1):14–20. 10.1002/iub.408 21280172

[47] Cen J, Wang M, Jiang G, Yin Y, Su Z, Tong L, et al The new immunosuppressant, isogarcinol, binds directly to its target enzyme calcineurin, unlike cyclosporin A and tacrolimus. Biochimie. 2015;111:119–24. 10.1016/j.biochi.2015.02.004 25701551

