# Supplementary material for: Prenylated Polyphenols from Clusiaceae and Calophyllaceae with Immunomodulatory Activity on Endothelial Cells
Source: PLoS One. 2016 Dec 1;11(12):e0167361. doi: 10.1371/journal.pone.0167361 (PMC5131938; doi:10.1371/journal.pone.0167361)
Supplement: S1 Table — (DOCX) [file pone.0167361.s001.docx]

**S1 Table. Purity (%) of NPs from the chemical library evaluated by HPLC equipped with ELSD and UV detectors**

|  | **Purity (%)** | | |
| --- | --- | --- | --- |
| **Compounds** | **210 nm** | **λ_max_** | **ELSD** |
| Coumarin **1** | 100 | 100 | Not detected |
| Umbelliferone **2** | 100 | 99.5 | 100 |
| Mammea A/AA **3** | 100 | 84.7 | 99.6 |
| Mammea A/AA cyclo F **4** | 100 | 97.5 | 100 |
| Neurophyllols B and A **5**-**6** | 100 / 0 | 83.0 / 17.0 | 92.8 / 7.2 |
| Mammea B/AB cyclo F **7** | Not detected | 92.8 | 100 |
| Lepidotol A **8** | 100 | 93.8 | 100 |
| Amentoflavone **9** | 97.4 | 100 | 100 |
| Blancoic acid **10** | 100 | 93.1 | 99.0 |
| Guttiferone J **11** | Not detected | 81.1 | 84.1 |
| Caloxanthone C **12** | 100 | 99.0 | 100 |
| Calothwaitesixanthone **13** | 100 | 99.0 | 100 |
