# Supplementary figures and images for: Prenylated Polyphenols from Clusiaceae and Calophyllaceae with Immunomodulatory Activity on Endothelial Cells
Source: PLoS One. 2016 Dec 1;11(12):e0167361. doi: 10.1371/journal.pone.0167361 (PMC5131938; doi:10.1371/journal.pone.0167361)

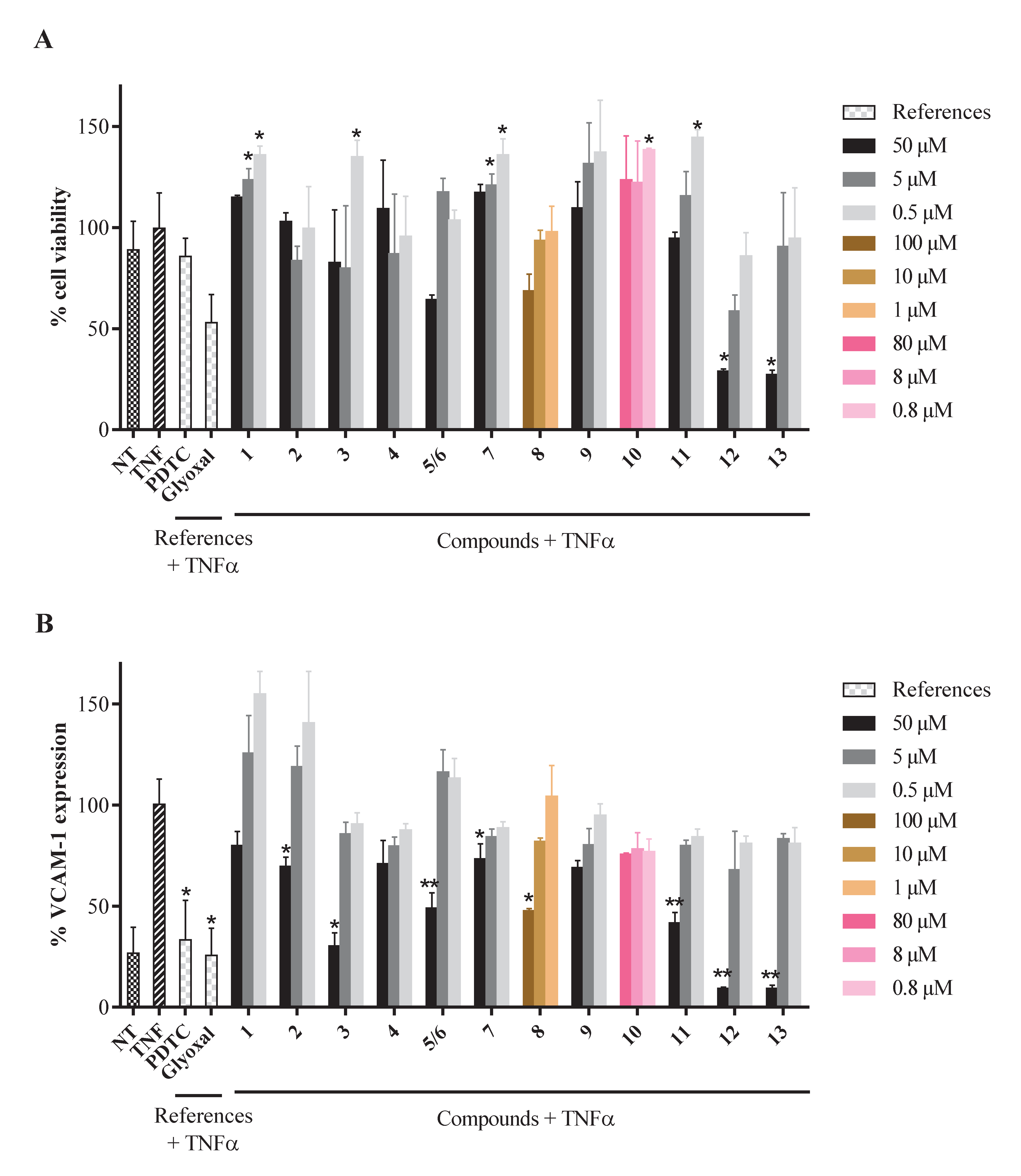

Supplement: S1 Fig — (A) Cellular toxicity on ECs was evaluated by a MTT assay using glyoxal (4 mM) as inducer of cell death control. (B) VCAM-1 surface expression was assessed by a cellular ELISA assay after activation of the cells by TNF (200 U/mL, 6h) and using pyrrolidine dithiocarbamate (PDTC, 200 μM) as an inhibitory reference. Statistical analysis of values (O.D.) obtained for treated versus non-treated cells (A) or TNF (B) were performed using non-parametric ANOVA test; (*p < 0.05, **p < 0.01 versus TNF). (TIF) [file pone.0167361.s002.tif]

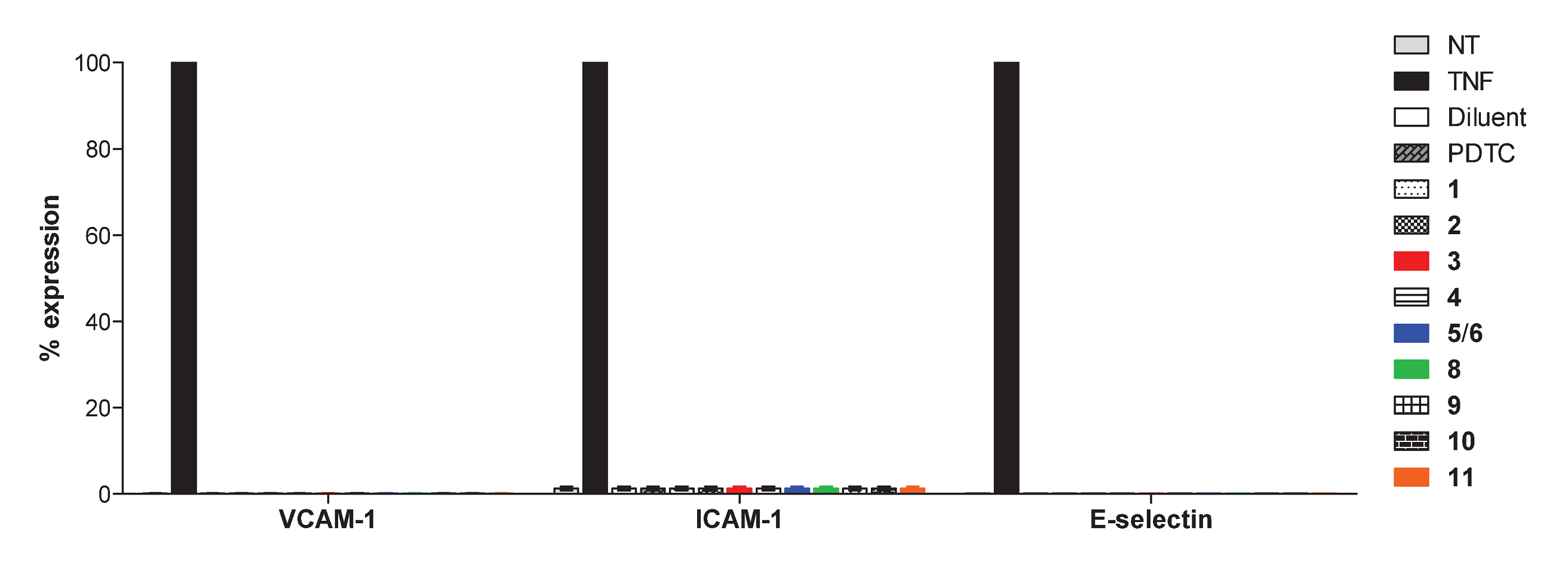

Supplement: S2 Fig — ECs were incubated with NPs (10 μM) for 6h in the absence of TNF. Cells were analyzed by facs for the expression of VCAM-1, ICAM-1 and E-selectin. TNF was used as a positive control. (TIF) [file pone.0167361.s003.tif]
